# Enhancing performance of Ag–ZnO–Ag UV photodetector by piezo-phototronic effect†

**DOI:** 10.1039/c8ra01189c

**Published:** 2018-04-23

**Authors:** Xiaotong Zhang, Yu Qiu, Dechao Yang, Bing Li, Heqiu Zhang, Lizhong Hu

**Affiliations:** School of Physics, Dalian University of Technology Dalian 116024 People's Republic of China yuqiu@dlut.edu.cn; Department of Electronic Engineering, Dalian Neusoft University of Information Dalian 116024 People's Republic of China; The Key Laboratory for Micro/Nano Technology and System of Liaoning Province, Dalian University of Technology Dalian 116024 People's Republic of China

## Abstract

In this work, an ultraviolet (UV) photodetector based on a ZnO nanowires (NWs) array with metal–semiconductor–metal Schottky junction structure was successfully fabricated on a flexible polyester fibre substrate by a low-temperature hydrothermal method. Subjected to a 0.2% tensile strain at −1 V, the *I*_light_ and sensitivity of the as-prepared UV photodetector are lifted by 82% and 130%, respectively. Furthermore, the response speed and recovery speed are significantly raised under the same tensile strain. The working principle can be explained as that the Schottky barrier height (SBH) is effectively improved by the negative strain-induced polarization at the metal–ZnO interface which is favorable for the separation of photogenerated electron–hole pairs. This work not only provides a facile and promising means to optimize the performance of a ZnO based MSM photodetector by applying a tensile strain but also opens up the way for fabrication and integration of ZnO photodetectors on flexible polyester fiber substrates.

## Abstract 2

An ultraviolet photodetector based on a ZnO nanowires with metal–semiconductor–metal Schottky structure was fabricated on a flexible polyester fibre substrate.

## Graphical abstract

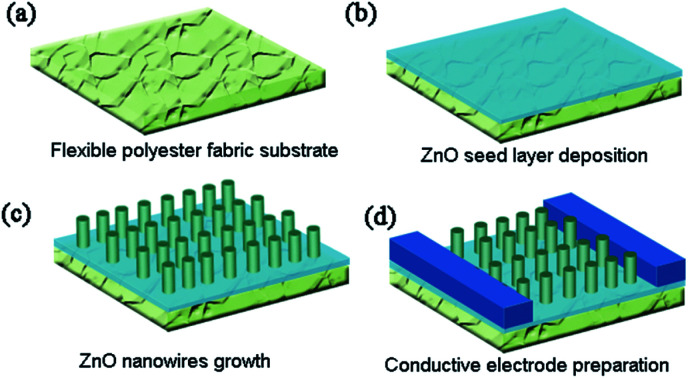

## 1. Introduction

Recently, ultraviolet photodetectors depending on diverse materials and mechanisms have been widely discussed in many fields such as solar-blind detectors, environment monitoring and chemical analysis.^1–3^ Among a variety of candidates, SiC,^4^ GaN^5,6^ and diamond^7^ are common semiconductors used for fabricating UV photodetectors. Compared with them, as a wide direct band-gap (3.37 eV) semiconductor, ZnO owns some unique merits to serve as a photo-absorber layer in a UV photodetector, for example, low price, simple synthesizing process,^8–10^ high response speed and abundant 1-D nanostructures. Hence, ZnO has attracted a great deal of interest and possesses the potential to be an alternative material in UV detectors.^11–16^ To improve the performance of ZnO based UV detectors, many materials such as PEDOT: PSS,^17^ Ag,^18,19^ ZnS,^20,21^ NiO^22,23^ have been employed to form a hetero-junction with ZnO. In terms of recent research, MSM structured Schottky junction UV detector formed by Ag and ZnO is extremely facile to be prepared which not only increases the photoresponsivity but also restricts the dark current. The low-cost, uncomplicated fabricating process supplies a possibility for realization of large-scale integration and mass production.

ZnO, a wurtzite-structured semiconductor with non-central symmetry, will generate a piezoelectric polarization under an external force. Based on the piezoelectric, semiconductor, phototronic three-way coupling characteristic, plenty of electronic devices have been developed including solar cells,^24^ light emitted diodes,^25^ photodetectors,^20,26^ strain sensors,^27^ field effect transistors^28^ and so on. For these junction-based devices, the transport, separation, recombination of charge carriers, barrier height and build-in electric field at the interface are modulated by strain-induced piezoelectric polarization.^27^ In respect of an Ag–ZnO Schottky junction UV photodetector, the depletion region at MS contact area provides a strong build-in field to separate electron–hole pairs under illumination. Therefore, according to the piezo-phototronics theory, the performance of the UV photodetector can be monitored and strengthened by external strain.^29^

Conventional UV photodetectors are usually manufactured on hard substrates like silicon, sapphire, ITO glass and all kinds of metal foil. With the revolution of science and technology, soft portable electronic devices which remarkably facilitate people's life have shown large scientific potential and commercial perspective.^30,31^ Therefore, it is a surge demand to develop electronic devices based on flexible, portable, wearable substrates. Recently, diverse soft substrates (like paper, flexible plastic and polymers) have been gradually applicated in variety of electronic devices, but these substrates also suffer from certain defects and need further research. For example, paper-based electronic devices are not durable enough and crispy. The plastic-based electronic devices are hard to integrate with wearable materials such as fiber and cloth. To our best knowledge, the fabrication of opto-electronic devices on fabric or fabric-like substrates is rarely reported. Therefore, the study about fabrication of components on fabric substrates can be novel and significant which may promote the development and integration of devices on wearable substrates.

In this paper, a ZnO MSM UV photodetector has been successfully synthesized on the flexible polyester fibre substrate by a low-temperature hydrothermal process. The detailed morphology and structural characterization are shown in the paper. More importantly, the UV photodetector can be further improved by piezo-phototronic effect under a small external strain. Basic mechanisms for observed characteristics are discussed in detail. This work not only studies the impact of deformation towards performance of Schottky-junction photodetector but also provides an effective method to optimize ZnO based MSM UV photodetector by a tensile strain. Furthermore, our work also broadens the road for the fabrication of photodetectors on flexible polyester fibers.

## 2. Experimental

### 2.1 Synthesis of ZnO NWs on polyester fibre substrate

For the construction of ZnO UV photodetector, we adopted commercially available polyester fibre as the substrate, which is a quite common material used everywhere in our daily life. Polyester fibre is born with massive merits serving as a substrate. For example, it is light-weight, wearable, portable, biocompatible and cheap.^32^ Firstly, the as-prepared substrates are cut into 1 cm × 1 cm pieces. Then, the pre-treated substrates are cleaned ultrasonically in turn by acetone, ethanol and deionized water for 15 minutes, respectively. After dried in heating oven for 1 hour at 80 °C, the polyester fiber substrates are well prepared. Before the growth of ZnO NWs, a 20 nm-thick ZnO seed layer is deposited on the polyester substrates with room temperature radio frequency magnetron sputtering. The background pressure of the vacuum chamber is 3 × 10^−4^ Pa and the pressure of the sputtering gas argon is 3.5 Pa. The radio frequency power is 140 W. Next, ZnO NWs are perpendicularly synthesized on the seed layer by hydrothermal method. The aqueous solution is composed of 30 mmol L^−1^ zinc acetate dehydrate and hexamethylenetetramine (molar ratio 1 : 1). Then, the seed layer substrates are kept vertically in a Teflon reaction kettle filled with solution and heated at 95 °C for 3 hours. After the reaction, the samples are rinsed for several times by deionized water and completely dried in heating oven.

### 2.2 Fabrication of Ag–ZnO–Ag UV photodetector

For the fabrication of ZnO UV photodetector, the ZnO NWs substrate is coated with two thin layers of Ag paste onto two ends of substrates to serve as electrodes. The distance between two electrodes is about 0.5 cm and the effective working area is about 0.5 × 1 cm^2^. Then both of Ag electrodes are connected with two Cu testing wires and dried in oven at 85 °C for half an hour. Finally, the whole device is successfully prepared.

### 2.3 Characterization

The crystal structure and surface morphology of ZnO NWs array are characterized by X-ray diffraction (XRD) and scanning electron microscope (SEM). Room temperature photoluminescence (PL) with a 325 nm He–Cd laser is carried out to perform the crystallographic quality of ZnO NWs. A Keithley 4200 semiconductor characterization system is used to measure photoelectric properties of the photodetector under a UV (365 nm) illumination (17 mW).

## 3. Results and discussion

### 3.1 Surface morphology and crystallinity

**Fig. 1:**
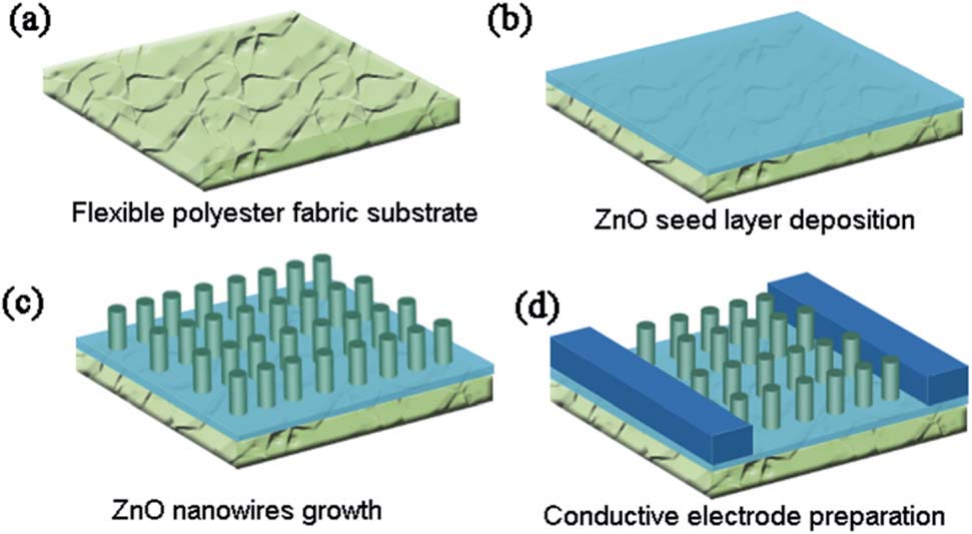
Schematic illustration of fabricating processes of Ag–ZnO–Ag photodetector: (a) polyester fabric substrate preparation, (b) ZnO seed layer deposition, (c) ZnO NWs growth, (d) conductive electrode preparation.

**Fig. 2:**
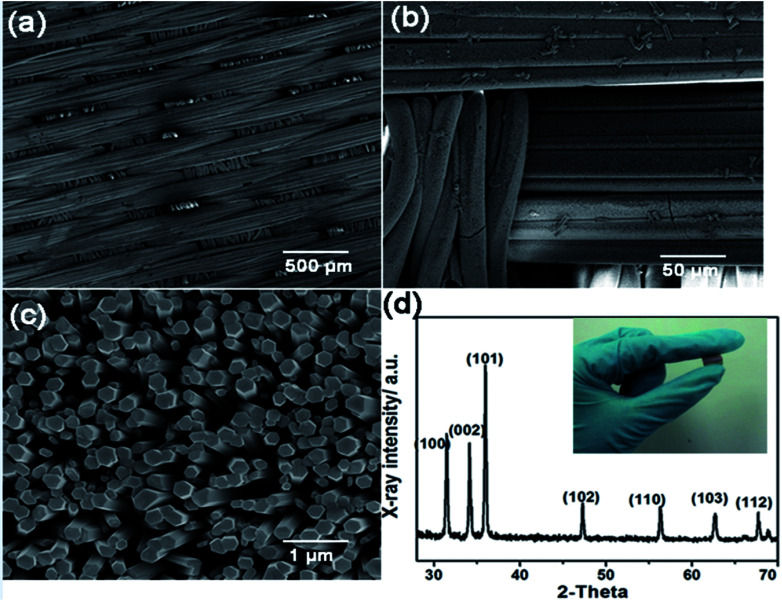
(a) and (b) Low-magnification SEM image of ZnO NWs on polyester fibre substrate. (c) High-magnification SEM image of ZnO NWs. (d) XRD pattern of ZnO NWs and optical image of as-fabricated device (inset).

The schematic diagram of the whole growth process is shown in Fig. 1, including ZnO seed layer deposition, ZnO NWs growth and conductive electrode preparation. The morphology images of ZnO NWs grown on the polyester fiber substrate under different magnifications are shown in Fig. 2(a–c). From Fig. 2(a) and (b), we can see that ZnO NWs are uniformly and densely covered onto polyester fibers. As shown in Fig. 2(c), all the ZnO NWs are in hexagonal prismatic structure with a diameter of 200–300 nm and a length of 4–5 μm. The well-aligned 1-D NWs have a clean and smooth top and side surfaces which not only contributes to the absorption of incident light from all directions but also is in favor of the formation of MSM Schottky junction.

The XRD patterns of ZnO NWs are utilized to characterize the crystal phase. As presented in Fig. 2(d), the (002), (100), (101) diffraction peaks are clearly observed which proves that the ZnO NWs have a hexagonal wurtzite structure and grow along *c*-axis. The XRD patterns also demonstrate the ZnO synthesized on polyester fibers exhibits a perfect crystalline quality and lays a good foundation for the performance of devices. The picture inside Fig. 2(d) indicated that the device is highly flexible and can be bent and fully recover. Fig. S1† also displays the room-temperature photoluminescence spectrum of prepared ZnO NWs sample. A near band edge (NBE) emission at 360 nm is obviously observed which results from the inter-band radiation combination of free excitons. Besides that, the broad, deep-level, yellow emissions also exist between 550–650 nm due to the impurity and defects of ZnO NWs such as oxygen vacancies. It should be noted that ZnO has high-density surface states which can lead to the Fermi-level Pinning Effect and surface band-bending phenomenon and the decrease of carriers' concentration. According to the theory mentioned above, although electron affinities of Ag and ZnO are nearly close, they can still compose a Schottky junction barrier. The barrier height is mainly dominated by the surface states rather than the work function.^28^

### 3.2 The performance of photodetector under UV illumination

To study the impact of piezo-phototronic effect on UV photodetector, a measuring setup is established. As shown in Fig. S2,† a plastic board (PB) which is about 20 cm long is fixed to a fixed sample platform at one edge and another edge is connected with a 3D portable mechanical stage. The PB is elastic and can be bent and curved with the movement of 3D mechanical stage. Our sample is mounted on the center of PB while the 365 nm UV illuminant is exactly above it. Considering that the device is much smaller than the PB, shifting the 3D mechanical stage down for 1 cm equals to subjecting a tensile strain to the photodetector.^29,30^ On the contrast, when shifting up the 3D stage, the photodetector receives a compressive strain. The deformation of photodetector *ε* can be calculated from *ε* = *d*/2*R*, where *d* is the up/down moving distance of 3D stage, *R* is the bending curvature. In this paper, while *d* is 1 cm and the length of PB is 20 cm, according to the formulation *h* = 2*R* sin^2^(*θ*/2) = 2*R* sin^2^(*L*/2*R*), the strain *ε* was calculated to be 0.2%.

**Fig. 3:**
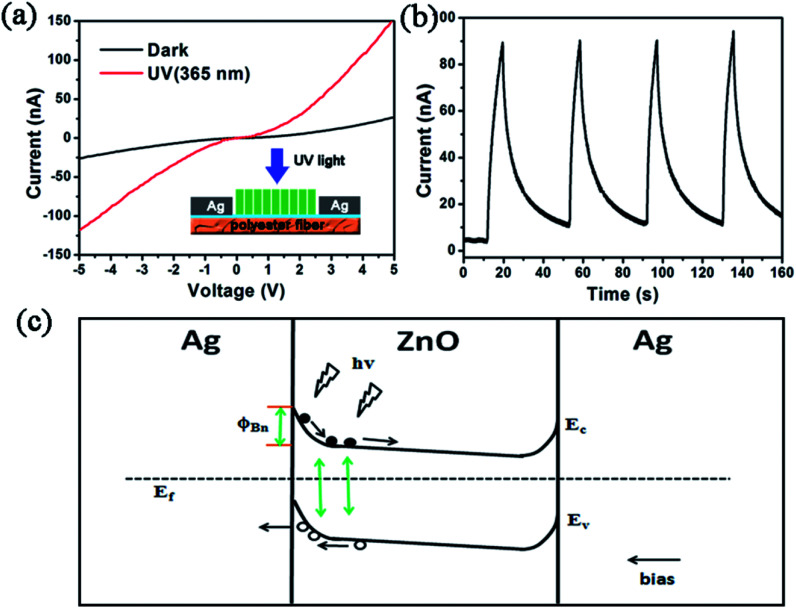
(a) The *I*–*V* characteristic curves in dark and under UV illumination. (b) The *I*–*T* characteristic curves of the ZnO UV photodetector at a bias of −4 V. (c) Schematic energy band diagram for illustrating the working principle of the UV photodetector under no strain.

Fig. 3(a) shows typical the *I*–*V* characteristic of Ag–ZnO–Ag UV photodetector on flexible polyester fibre substrate in the dark (black) and under UV illumination (365 nm, 0.5 μW cm^−2^). The absolute current is improved from 25 nA (dark current) to 150 nA at a bias of 5 V meanwhile the photocurrent is about 6 times higher than the dark current. The sensitivity which is defined as (*I*_light_ − *I*_dark_)/*I*_dark_ is as much as 5 here. Fig. 3(b) depicts the photocurrent of the as-fabricated ZnO UV photodetector under illumination at a bias of −4 V. The photocurrent–time curve demonstrates that the device has a rapid response speed and a fast recovery speed. Moreover, after many times' repeated cycles, the photocurrent doesn't drift and can fully recover which proves our sensors have a good stability and repeatability.

The symmetric rectifying *I*–*V* characteristics with and without illumination indicate that two back to back Schottky junctions based on Ag–ZnO–Ag structure have been successfully fabricated. On one hand, the MSM design can dramatically suppress the dark current thereby increasing the sensitivity; on the other hand it could monitor the separation and transport of carriers through the Schottky junction. When a constant voltage bias is applied on the photodetector, the voltage drop mainly locates at the reversely biased Schottky junction,^33,34^ therefore, the photoelectric property of the device is chiefly dominated by the reversely biased Schottky junction. Hence, in dark condition, the current should be expressed in terms of thermionic-emission theory as follows:1
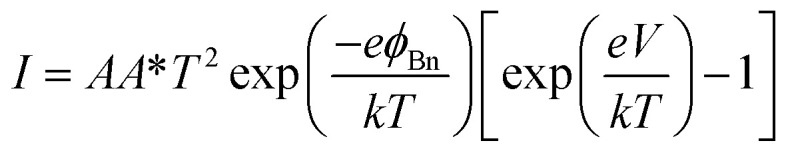
where *A* is the area of the contact, A* is Richardson constant, *T* is the Kelvin temperature, *e* is the charge of electron, *ϕ*_Bn_ refers to the height of Schottky junction barrier, *k* is the Boltzmann constant, *V* represents the external bias.

As to the photocurrent, the situation can be discussed in two aspects. Outside the contact area, NWs will absorb the oxygen molecules from atmosphere and the O_2_ will capture electrons from ZnO which brings about the formation of a large quantity of holes distributing among the surface in dark. Then the free holes near surface and the free electrons from inner part construct a depletion region which decreases the conductivity of ZnO NWs.^35^ When the detector is exposed to UV illumination whose energy is greater than the bandgap of ZnO, electron–hole pairs are generated along the NWs. The photo-generated holes compensate with trapped electrons then oxygen molecules are desorbed into the air. At the same time, the unpaired photo-generated electrons are released into internal NWs and lift the conductivity of photodetector.^36,37^ When considering about the junction area, we have noticed that half proportion of the device belongs to the junction area and the characteristic of Schottky junction is relatively remarkable. As a consequence, according to the energy band schematic diagram in Fig. 3(c), when illumination falls on the sensor, electron–hole pairs are generated at two heterojunctions. At the reversely biased junction area, the generated holes flow to the Ag electrode and the generated electrons drift to the ZnO NWs array.^15^ Nevertheless, the photocurrent produced at forward biased Schottky junction is seriously confined by the reversely biased junction, so we won't take the current generated at forward biased junction into consideration. Then the photocurrent is created by the cooperated effect inside and outside the junction area. With regard to the phenomenon that the recovery time is longer than the response time presented in Fig. 3(b), the reason can be explained as that after the illumination is withdrawn, the ZnO NWs absorb oxygen molecules again followed by the decrease of conductivity. This process takes more time so the recovery speed is a bit lower.

### 3.3 Piezoelectric effect on the performance of UV photodetector

**Fig. 4:**
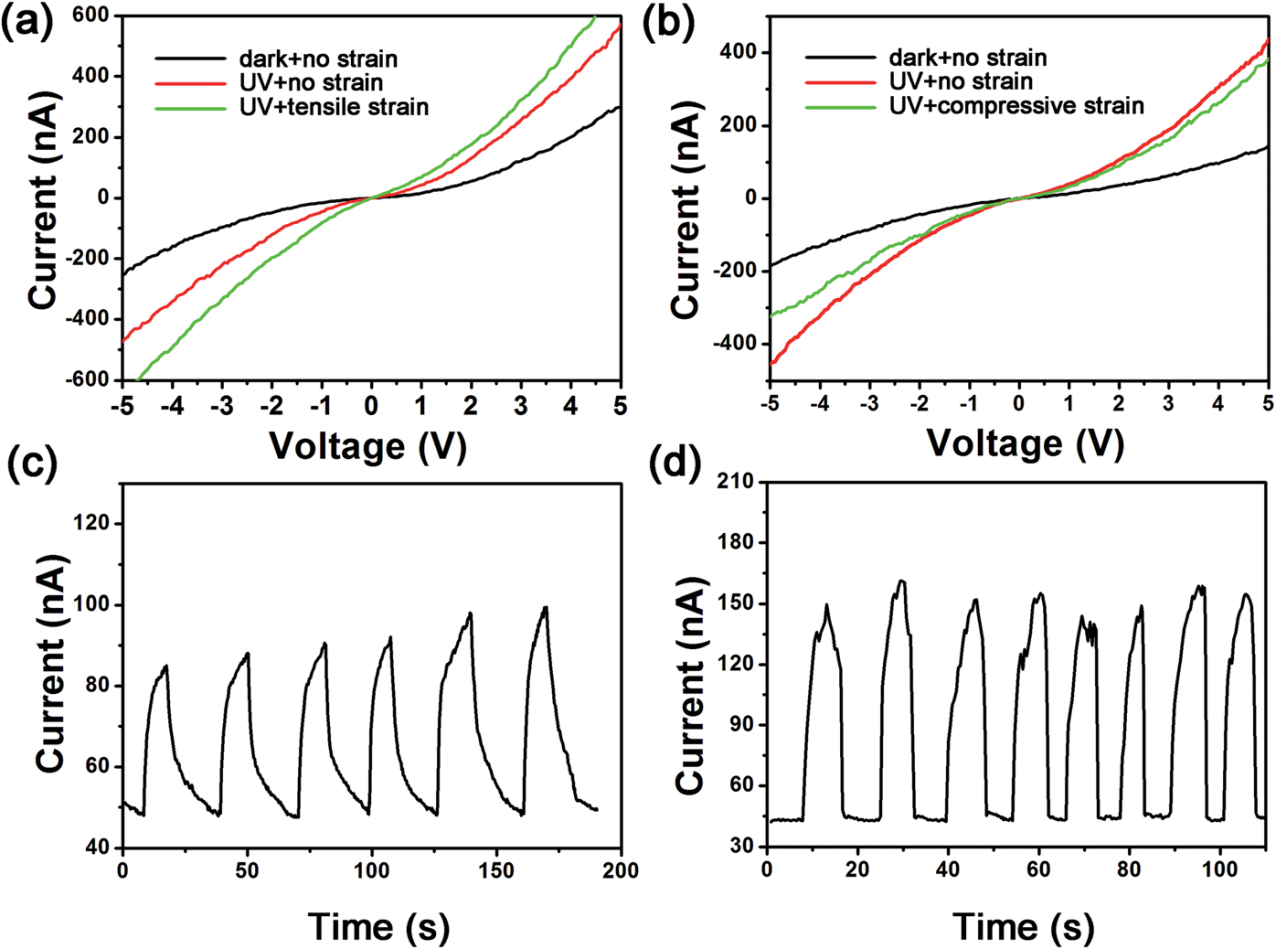
(a) The *I*–*V* characteristic curves with/without tensile strain and with/without UV light. (b) The *I*–*V* characteristic curves with/without compressive strain and with/without UV light. (c) Time dependence of the photocurrent without external strain at a bias of −1.5 V. (d) Time dependence of the photocurrent with tensile strain at a bias of −1.5 V.

Fig. 4 has illustrated the influence of external strain on the performance of UV photodetector. The *I*–*V* characteristics under tensile strain and compressive strain with and without UV light measured at a bias of −5 V to +5 V are described in Fig. 4(a) and (b). As displayed in Fig. 4(a), at biases of −1 V, −2 V and −3 V and under a 0.2% tensile strain, the absolute photocurrents increase from 44.3 nA, 120.3 nA, 221.1 nA to 80.8 nA, 196.5 nA, 331.9 nA and the sensitivities improve from 1.72, 1.47, 1.29 to 3.96, 3.03, 2.45, respectively. On the contrary, from Fig. 4(b), we can see that both of the photocurrent and sensitivity decline under compressive strain. For example, at a bias of −3 V, the absolute photocurrent and sensitivity reduce from 211.2 nA and 1.49 to 173.5 nA and 1.05 as a function of a 0.2% compressive strain. In addition, the photocurrent–time curves at a bias of −1.5 V under no-strain condition and under a 0.2% tensile strain are depicted in Fig. 4(c) and (d). As shown in the diagram, the average peak photocurrent is lifted from 92 nA to 153 nA under the tensile strain. Moreover, the average time of photocurrent responding to peak value and the average time of photocurrent recovering to initial value are decreased from 11.3 s and 18.8 s to 4.4 s and 1.9 s. Through comparing two *I*–*T* characteristics with and without tensile strain, a conclusion has been summarized that tensile strain can not only improve the photocurrent but also promote the response speed and recovery speed.

**Fig. 5:**
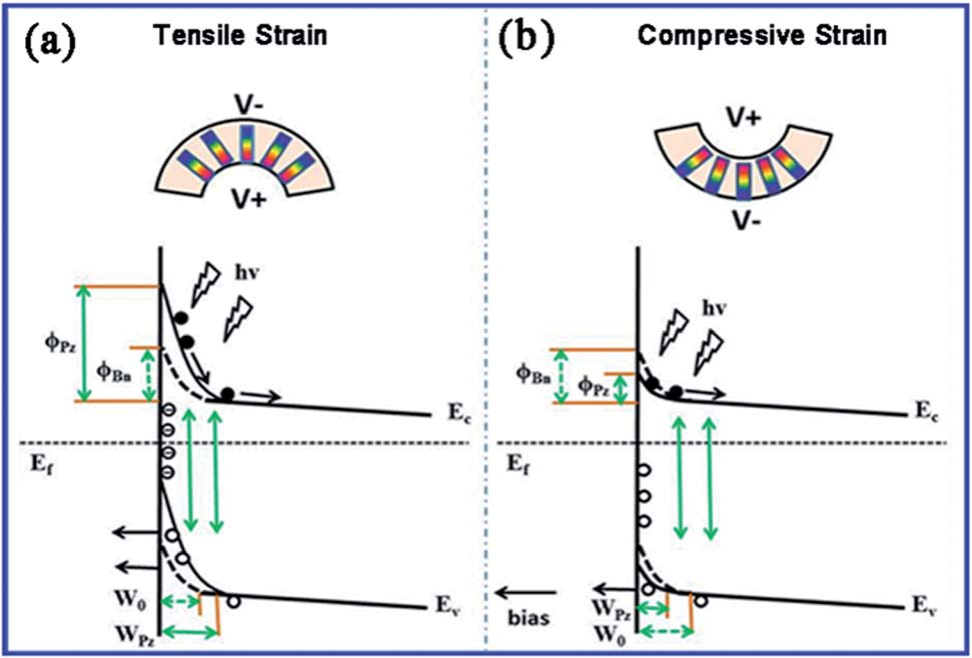
Schematics and energy band diagrams for illustrating the working principle of the UV photodetector under (a) tensile strain and (b) compressive strain.

The enhancing performance of Ag–ZnO–Ag UV photodetector by piezo-phototronic effect can be explained from the principle proposed in Fig. 5. Fig. 5(a) and (b) are schematic diagrams of energy band with tensile strain and compressive strain under UV illumination. When a tensile strain is applied to the device, a negative piezo-polarization will be formed at the interface of Ag and ZnO. The strain-induced negative piezo-polarization charges gathering at the junction area elevate the Schottky barrier height (SBH) as shown in Fig. 5(a) and the variation of SBH caused by piezoelectric effect can be simplified as:^38^2
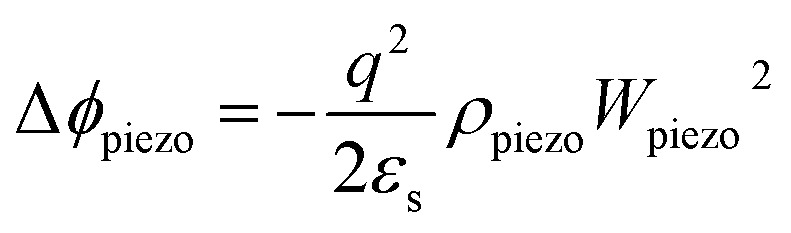
where *ε*_s_ is relative dielectric constant, *q* is the charge of the electron, *ρ*_piezo_ represents the density of piezoelectric charges and *W*_piezo_ refers to the width of piezo-charges. In addition, under the tensile strain, the width of depletion region is broadened as well on account of the negative piezo-charges and can be described as following:3
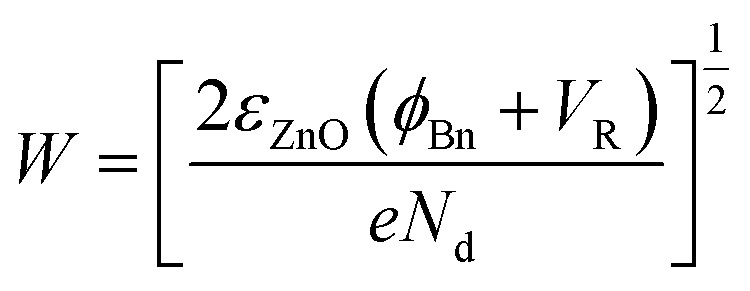
where *ε*_ZnO_ is the dielectric constant of ZnO, *V*_R_ is the reversely voltage bias, *ϕ*_Bn_ is the Schottky junction barrier height that can be enhanced by external strain and *N*_d_ is the density of the electrons in ZnO. As illustrated in Fig. 5(a), depletion width is broadened from *W*_0_ to *W*_pz_ which increases monotonically with the SBH.4
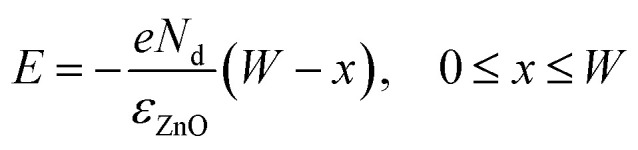


Therefore, from eqn (4) we can conclude that the build-in electric field is strengthened by the enhanced SBH and depletion width. Overall, the enhanced Schottky barrier height, depletion region width, and build-in field are beneficial for the separation of photogenerated electron–hole pairs, thus a higher photocurrent, sensitivity and a rapider response, recovery speed are obtained. This is the principle of the Ag–ZnO–Ag UV photodetector enhancing performance by piezo-phototronic effect. Inversely, a positive piezo-polarization is induced at the Ag/ZnO interface by compressive strain which decreases the SBH, depletion width, build-in field and weakens the separation of photogenerated electron–hole pairs. Then the photocurrent and sensitivity are reduced. This work also provides a promising approach to modulate the property of ZnO based photosensors and evidence the feasibility of flexible polyester fibers applicated in piezo-photoelectronic devices.

## 4. Conclusion

In summary, we have successfully fabricated the Ag–ZnO–Ag UV photodetector based on ZnO NWs by a simple low-temperature hydrothermal method. On account of the unique piezo-semiconductor property of ZnO, the performance of UV photodetector can be optimized by an external strain. Under a 0.2% tensile strain, the photocurrent and sensitivity are raised by 82% and 130% at a bias of −1 V. Moreover, at a bias of −1.5 V, the response speed and recovery speed are significantly improved at the same strain. The result shows that the negative piezo-polarization charges induced by tensile strain increase the Schottky junction barrier height, depletion region width and build-in electric field, which is favorable for the separation of photogenerated electron–hole pairs. This work broadens the road for integration of electronic devices on flexible polyester fibers and offers a facile means to enhance the performance of ZnO based photodetectors by piezo-phototronic effect.

## Conflicts of interest

There are no conflicts to declare.

## Supplementary Material

RA-008-C8RA01189C-s001
