# Supplementary material for: Enhancing performance of Ag–ZnO–Ag UV photodetector by piezo-phototronic effect
Source: RSC Adv. 2018 Apr 23;8(28):15290–6. doi: 10.1039/c8ra01189c (PMC9082044; doi:10.1039/c8ra01189c)
Supplement: RA-008-C8RA01189C-s001 [file RA-008-C8RA01189C-s001.pdf]

Electronic Supplementary Material (ESI) for RSC Advances.
This journal is © The Royal Society of Chemistry 2018

Supporting Information

Enhancing performance of Ag-ZnO-Ag UV photodetector by piezo-phototronics effect

Xiaotong Zhang[1], Yu Qiu[1,3,*], Dechao Yang[2], Bing Li[1], Heqiu Zhang[1,3], Lizhong Hu[1,3,*]

[1]School of Physics and Optoelectronic Technology, Dalian University of Technology, Dalian 116024, People's Republic of China

[2]Department of Electronic Engineering, Dalian Neusoft University of Information, Dalian, 116024, People's Republic of China

[3]The Key Laboratory for Micro/Nano Technology and System of Liaoning Province, Dalian University of Technology, Dalian 116024, People's Republic of China

*Corresponding Author. E-mail: yuqiu@dlut.edu.cn,

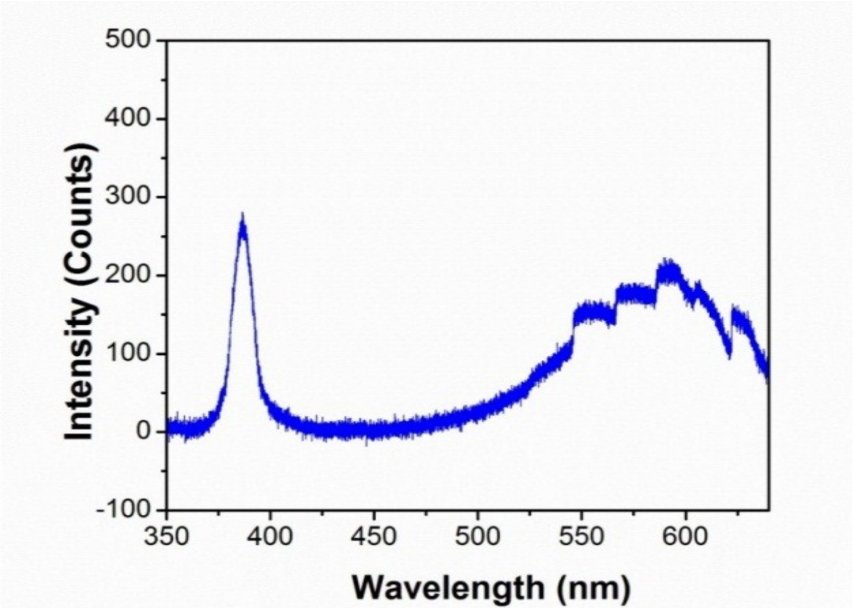

Figure S1 Room-temperature photoluminescence spectrum of as-grown ZnO nanowires.

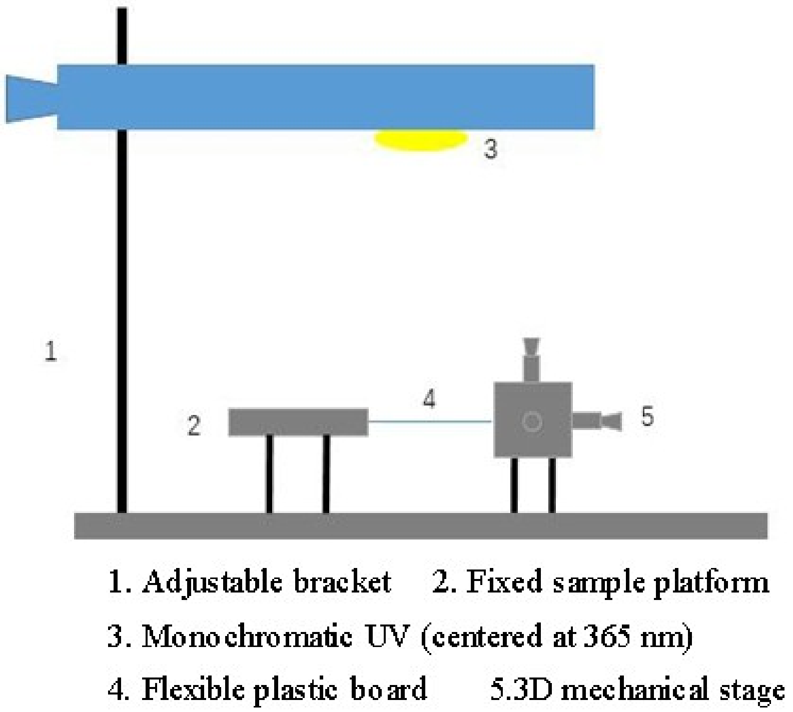

Figure S2 . Schematic diagram of the measuring setup.
